# Women as Physicians

**Published:** 1880-04

**Authors:** 


### WOMEN AS PHYSICIANS.

IN an article in the *International Review*, Dr. Chadwick makes the just observation that the question is no longer, "Shall women be allowed to *practice* medicine?" They are practicing it, not by ones and twos, but by hundreds; and the only problem now is, "Shall we give them opportunities for studying medicine before they avail themselves of the already acquired right of practicing it?" It is clearly the interest of the community to give to women the fullest instruction, in accordance with the most improved systems, and under the most eminent teachers; and also that their proficiency should be tested by the most rigid ordeals before they finally receive certificates. By a recognition of these certificates and their comparative values, the community would be able to protect itself from the impositions of ignorant or fraudulent pretenders to medical knowledge.

